# Fortunate Finger Deviation of a Fatal Heart Bullet Injury

**DOI:** 10.4103/1995-705X.76810

**Published:** 2010

**Authors:** Duilio Divisi, Carmine Villani, Sergio Di Tommaso, Alessandro Mazzola

**Affiliations:** Department of Cardiothoracic Surgery, “G. Mazzini” Hospital, Teramo, Italy

**Keywords:** Computerized tomography, firearm heart lesion, surgical treatment

**Figure 1:**
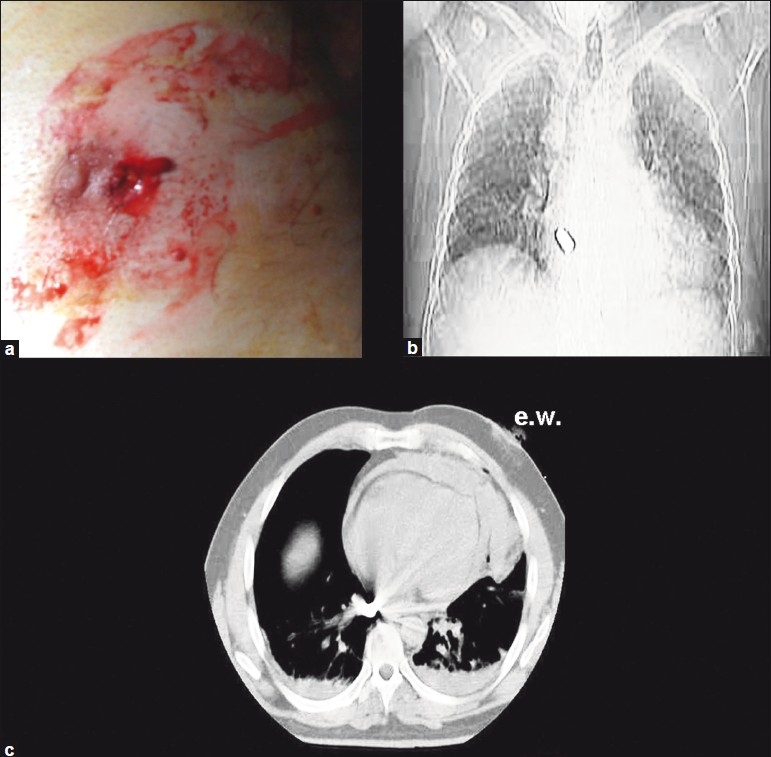
a) The bullets left nipple line wound; b) chest X-ray shows the intracardiac bullet; c) CT of the thorax reveals bilateral hemothorax and a huge fluid collection in the pericardium. The ribs were undamaged. The trajectory of the bullet was deviated by the finger seems to be from the left to the right side of chest

**Figure 2:**
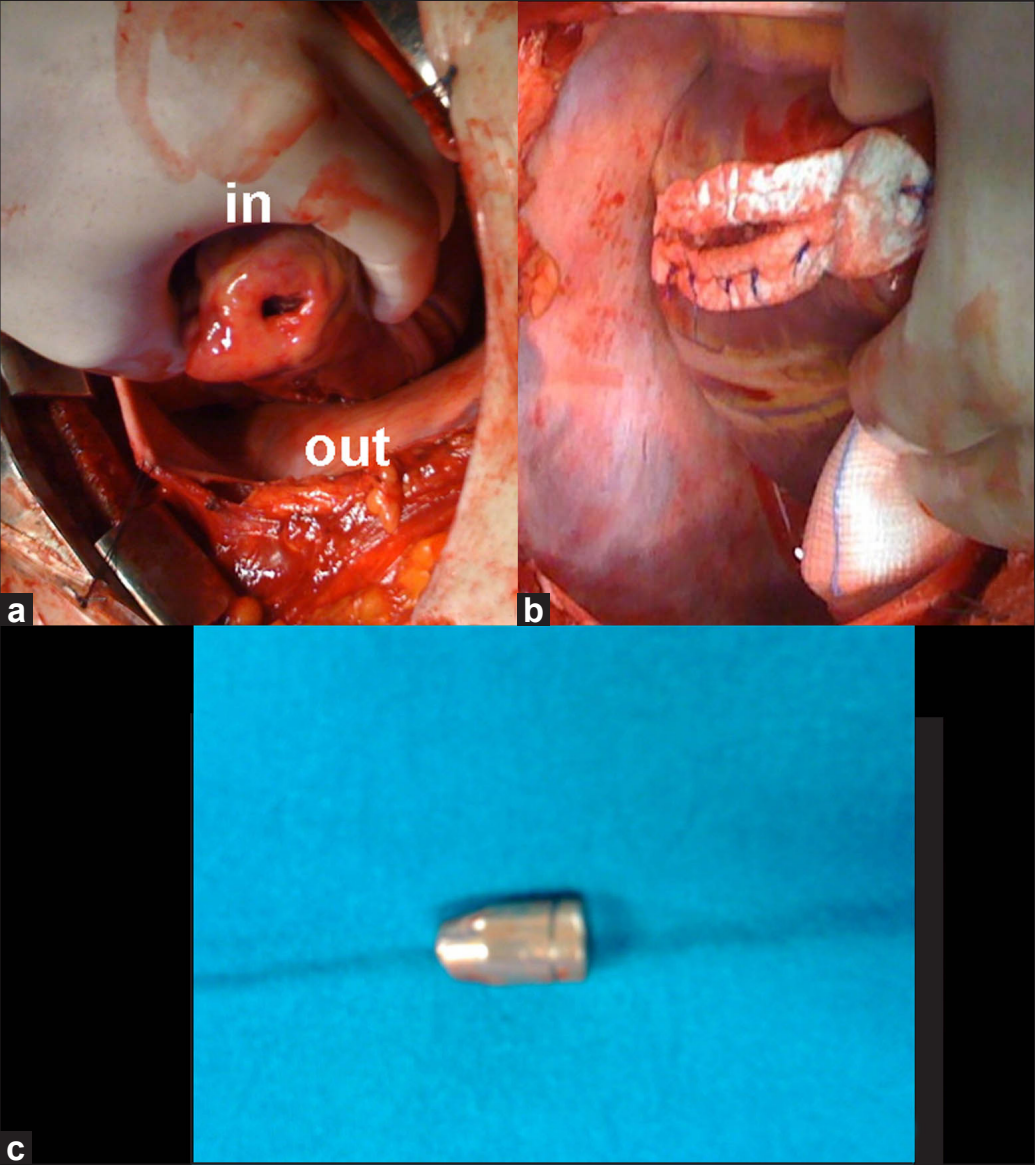
Intraoperative view. a) The entry and exit heart wounds; b) the reinforcement of suture by Teflon strips; c) bullet

A young man arrived at the emergency room complaining of two lesions: one in the middle left finger and the other in the left nipple line. He was hemodynamically stable, conscious and able to say that he had been shot at a distance of 3 m, protecting himself with his left hand. Based on computerized tomography (CT) of the thorax [Figure 1], we preferred median sternotomy approach instead of videothoracoscopy.[1,2] We discovered a left ventricular injury without rupture of cardiac cavity; the bullet was found in the pericardium. The heart bullet injury was repaired with interrupted monofilament stitches reinforced by Teflon strips [Figure 2].[3]
